# Efficient Algorithm for Mining Non-Redundant High-Utility Association Rules

**DOI:** 10.3390/s20041078

**Published:** 2020-02-17

**Authors:** Thang Mai, Loan T.T. Nguyen, Bay Vo, Unil Yun, Tzung-Pei Hong

**Affiliations:** 1 Institute of Research and Development, Duy Tan University, Da Nang 550000, Vietnam; mhthang.it@gmail.com or; 2 School of Computer Science and Engineering, International University, Ho Chi Minh City 700000, Vietnam; 3 Vietnam National University, Ho Chi Minh City 700000, Vietnam; 4 Faculty of Information Technology, Ho Chi Minh City University of Technology (HUTECH), Ho Chi Minh City 700000, Vietnam; 5 Department of Computer Engineering, Sejong University, Seoul 05006, Korea; yunei@sejong.ac.kr; 6 Department of Computer Science and Information Engineering, National University of Kaohsiung, Kaohsiung 811, Taiwan; tphong@nuk.edu.tw; 7 Department of Computer Science and Engineering, National Sun Yat-sen University, Kaohsiung 804, Taiwan

**Keywords:** data mining, non-redundant high-utility association rule, high-utility association rule, high-utility itemset, lattice, Internet of Things

## Abstract

In business, managers may use the association information among products to define promotion and competitive strategies. The mining of high-utility association rules (HARs) from high-utility itemsets enables users to select their own weights for rules, based either on the utility or confidence values. This approach also provides more information, which can help managers to make better decisions. Some efficient methods for mining HARs have been developed in recent years. However, in some decision-support systems, users only need to mine a smallest set of HARs for efficient use. Therefore, this paper proposes a method for the efficient mining of non-redundant high-utility association rules (NR-HARs). We first build a semi-lattice of mined high-utility itemsets, and then identify closed and generator itemsets within this. Following this, an efficient algorithm is developed for generating rules from the built lattice. This new approach was verified on different types of datasets to demonstrate that it has a faster runtime and does not require more memory than existing methods. The proposed algorithm can be integrated with a variety of applications and would combine well with external systems, such as the Internet of Things (IoT) and distributed computer systems. Many companies have been applying IoT and such computing systems into their business activities, monitoring data or decision-making. The data can be sent into the system continuously through the IoT or any other information system. Selecting an appropriate and fast approach helps management to visualize customer needs as well as make more timely decisions on business strategy.

## 1. Introduction

In recent years, the Internet of Things (IoT) has offered many useful applications in healthcare, transportation, agriculture, trade, etc. For instance, in retail groceries, IoT has built an infrastructure to enable real-time interaction with customers in both physical and virtual stores [1]; Bluetooth-based positioning systems have been combined with processing mining to investigate customer behaviors regarding gender using their paths through a shopping mall [2]. Applying IoT into business brings great value and convenience to management. However, with the growth of IoT, a large number of transactions are being tracked continuously, and thus there is a massive amount of data. Efficient methods of data mining are necessary for better decision-making in this context. Many researchers have focused on investigating data mining solutions for IoT and sensor systems, including works such as: “Detecting Incremental Frequent Subgraph Patterns in IoT Environments” [3], “Mining Productive-Associated Periodic-Frequent Patterns in Body Sensor Data for Smart Home Care” [4], and “Mining Algorithm for Discovering Sequential Pattern in Wireless Sensor Network Environments” [5].

In traditional approaches [6], the mining of association rules is based on a support-confidence framework in which the items have no difference in terms of importance between transactions, and the utility (e.g., the weight) of each item is not taken into consideration. In recent decades, many researchers have dedicated their efforts to high-utility itemset (HUI) mining, and several publications have been released, for example on the mining of HUIs from vertically distributed databases [7], a two-phase algorithm [8], the HUI-Miner algorithm, which uses a utility-list structure to store both utility and heuristic information for pruning the search space [9], the EFIM algorithm, with an effective search space pruning strategy [10], the mining of the top-k HUIs [11], and mining of HUIs with multiple minimum utility thresholds [12]. In HUI mining (HUIM), each item is associated with a weight (e.g., a utility, profit, etc.). An itemset is called a HUI if its utility is greater than a specified minimum utility threshold (*min-util*).

HUIM is a more difficult problem than frequent itemset mining (FIM) [13] since the downward-closure property does not hold. This property states that the subsets of a frequent itemset are frequent, and that the supersets of an infrequent itemset are infrequent (and therefore anti-monotonic). This has formed the basis for various previously developed methods, and is used to discard the redundant parts of the search space. The supersets and subsets of a HUI may have a utility that is lower, equal to, or higher than the utility of the itemset, and thus the utility of an itemset cannot satisfy the downward-closure property. Many recent algorithms for mining HUIs have focused on reducing the number of candidates generated [8,9,10,14]. However, relatively little effort has been made to generate rules from HUIs.

Sahoo et al. [15] developed a method to mine HARs from HUIs. For each high-utility closed itemset (HUCI), the algorithm scans all generators of each subset of HUCIs to generate a high-utility generic basis (HGB), and from the HGB the authors proposed an algorithm to generate all HARs. Mai et al. [16] also introduced the LARM algorithm by applying a lattice structure to construct a semi-lattice of HUIs, and then to generate all HARs.

### 1.1. Motivation

Sahoo et al. [15] and Mai et al. [16] focused on the problem of mining all HARs. In some decision-support systems, users only need to mine a small set of HARs for efficient use. Therefore, this paper proposes a method for the efficient mining of NR-HARs. This approach can be applied into any decision-making system in order to increase its potential to support business decisions based on a set of NR-HARs instead of large set of HARs.

### 1.2. Contributions

The primary contributions of this research to a solution for mining NR-HARs using an HUI semi-lattice structure are as follows:

- We provide a complete definition of NR-HARs based on HGB [15] in order to follow the best practice of association rule mining that the rules should follow a condition related to their confidence.
- We propose the LNR-HAR algorithm generating all NR-HARs based on a lattice of HUIs.
- We experiment with various conditions to explore the efficiency of the LNR-HAR algorithm so that this algorithm will be the best choice applying into any real applications, which need to generate NR-HARs.

The remaining sections are organized as follows. We review related works on mining HUIs, HARs, and association rules from a lattice in Section 2. In Section 3, we present a statement of the problem and some basic definitions, along with some theorems and propositions, where possible. The new algorithm, LNR-HAR, is developed and illustrated in Section 4. Section 5 describes the performance evaluation of the algorithm in detail, based on experimental results for both the runtime and memory utilization. In the final part, we present the conclusion and discuss some directions for future research.

## 2. Related Work

### 2.1. High-Utility Itemset Mining

It is easy to recognize that each item has its own weight in a real transaction database, and many recent studies take the utility into consideration. The purpose of HUIM is to solve problems that FIM cannot. In HUIM, each item can appear one or many times in each transaction, and each item has its own weight.

Several algorithms for HUIM have been proposed. Liu et al. [8] designed an algorithm for mining HUIs, although this needs to scan the database multiple times and generates many candidates. Many approaches have thus been put forward to avoid large numbers of database scans and the generation of numerous candidates, such as those using the incremental high-utility pattern (IHUP) [17], fast high-utility miner (FHM) [18], efficient high-utility itemset mining (EFIM) [10], high-utility itemset miner (HMiner) [19], utility-list buffer for high-utility itemset miner (ULB-Miner) [20], and sliding window based high-utility pattern mining (SHUPM) [21].

Ahmed et al. [22] also investigated the HUIM problem and proposed a new approach for mining HUIs by applying new three novel tree structures: (1) incremental HUP lexicographic tree (IHUPL-Tree) to capture the incremental data without any restructuring operation; (2) IHUP transaction frequency tree (IHUPTF-Tree) to obtain a compact size by arranging items according to their transaction frequency (descending order); and (3) IHUP-transaction-weighted utilization tree (IHUPTWU-Tree), which is designed based on the TWU value of items in descending order to reduce the mining time.

Fournier-Viger et al. [18] presented the FHM algorithm, which is considered effective for mining HUIs; however, this approach encounters problems with storage space due to the generation of a huge set of HUIs. Zida et al. [10] investigated the calculation of a new and tighter upper bound on the utility, called the sub-tree utility, at parent nodes rather than at child nodes during a depth-first search, and proposed the EFIM algorithm; this is a very effective algorithm, which effectively prunes the redundant search space.

Krishnamoorthy [19] examined the utility list and introduced a novel compact version, and also defined a data structure called a virtual hyperlink. This new algorithm and data structure were applied in the HMiner algorithm in combination with a candidate pruning strategy to give an effective technique for mining HUIs. Duong et al. [20] realized that using a utility list structure in the mining of HUIs involves high memory consumption. These authors proposed the ULB-Miner algorithm, which uses a novel structure called a buffered utility list to reuse the memory allocation for the utility list, so that the set of HUIs can be returned quickly without the need for a large amount of memory. Yun et al. [21] investigated the HUIM problem and proposed a representative algorithm called SHUPM. This new solution does not generate candidate itemsets, and hence reduces the search space so that the HUIM process is more effective in terms of runtime and memory usage. Nguyen et al. [14] proposed an efficient method for mining HUI in dynamic profit databases. The authors stated the problem of mining HUIs in such databases, modified the EFIM algorithm as a baseline algorithm, and proposed an efficient algorithm based on P-set to reduce the number of transaction scans.

Song and Huang [23] proposed a HUIM framework, (Bio-HUIF) including three algorithms, HUIF-PSO, HUIF-GA, and HUIF-BA, for mining high-utility itemsets in a transaction database using evolutionary algorithms (bioinspired algorithms). The authors also conducted multiple experiments and found that it performed better in term of speed compared to the HUPEUMU-GARM algorithm (GA-based HUIM algorithm) proposed by Kannimuthu and Premalatha [22] and HUIM-BPSO algorithm developed by Lin et al. [24].

Recently, Dawar et al. [25] proposed a hybrid framework for mining HUIs. This algorithm is more effective than the FHM [18] and EFIM algorithms [10], especially on dense datasets.

Most researchers have been focusing on how to eliminate redundant candidates during the process of mining HUIs; however, the time needed to compute the utility value of itemsets is a significant part of the whole running time. To reduce the long runtime of utility computation, Qu et al. [26] thus presented the basic identification algorithm (BIA) for mining HUIs. They then proposed a candidate tree with a novel structure and based on this developed a candidate tree-based algorithm called the fast identification algorithm (FIA) to quickly identify HUIs. The FIA is able to mine HUIs quickly, although it consumes much memory since it is based on a candidate tree, which is saved completely in the memory.

Wu et al. [27] also investigated the same problem and found that most of previous algorithms generate large set of candidates. They then proposed a new approach which applied pruning strategies and named the algorithm HUI-PR (HUIM with pruning strategies). The algorithm aimed to reduce the computation time as well as reduce the search space.

Gan et al. [28] extended the occupancy measure to assess the utility of patterns in transaction databases. The authors then proposed the high-utility occupancy pattern mining (HUOPM) algorithm. This focuses on user preferences in terms of frequency, utility, and occupancy. They also presented a novel frequency-utility tree (FU-Tree) and new kinds of compact data structures (utility occupancy list and FU-table). Based on these, HUOPM can extract complete HUIs quickly without candidate generation.

Recently, Gan et al. [29] conducted a survey of utility-oriented pattern mining (UPM) to present current approaches to high-utility itemset mining. The authors reviewed the current basic approaches, including apriori-based approaches, tree-based approaches, projection-based pattern-growth approaches, and new data format-based approaches. They then did surveys on some advanced topics of HUIM, such as mining high average utility itemsets, HUIM in dynamic environments, concise representations of utility patterns, mining high-utility quantitative itemsets and rules, high-utility sequential pattern mining, high-utility episode mining, UPM in big data, UPM in stream data, and UPM with various interesting constraints.

Besides high-utility itemsets mining, researchers have also focused on an extension problem of high-utility itemset mining, i.e., high average utility itemset mining. Because the utility of a larger itemset is generally greater than that of a smaller itemset, high average utility itemsets provide a better assessment of the utility of each itemset by considering both the length of itemsets and their utilities. The HAUI-Miner algorithm proposed by Lin et al. [30], a more efficient algorithm with multiple minimum high average-utility counts (called MEMU) proposed by Lin et al. [31], and the HAUIM algorithm proposed by Zhang et al. [32], are representative approaches to solving high average utility itemset problems.

### 2.2. High-Utility Association Rule Mining

The utility-confidence framework is widely used in multiple systems and applications, and especially in retail and e-commerce. Lee et al. [33] proved this in their research on marketing solutions for cross-selling using utility-based association rule mining as compared to HUIM.

Sahoo et al. [15] presented a definition of NR-HARs, and proposed the HGB algorithm to generate NR-HARs and the HGB-HAR algorithm to explore a set of high-utility association rules (HARs).

Mai et al. [16] also applied the lattice concept [34] to mine HARs better. Their approach [35] involved constructing a lattice structure of HUIs, and then using the LARM algorithm to generate HARs. The lattice approach was proved to be efficient for mining frequently closed itemsets [34] and closed high-utility itemsets [36], mining generalized association rules [37], mining non-redundant association rules [38], and mining association rules [39]. With regard to HUIM, LARM is the first algorithm that applies the lattice approach and it has shown good performance in the mining of HARs, especially when used on large datasets.

## 3. Problem Statement

Let *I* be a finite set of items, *I =* {*i*_1_, *i*_2_, …, *i_m_*}, in which each item *i_r_* ∈ *I* (1 ≤ *r* ≤ *m*) has a weight (utility) value *p*(*i_r_*). Itemset *X* is formed from a collection of *k* distinct items, *X* = {*j*_1_, *j*_2_, …, *j_k_*} (*j_u_* ∈ *I*). A set of transactions *T_id_* (where *id* is a unique identifier) is called a database *D* = {*T*_1_, *T*_2_, …, *T_n_*}. In each different transaction *T_id_*, each item *i_r_* is associated with a quantity value *q*(*i_r_*, *T_id_*), which is the number of items purchased *i_r_*. The problem of mining all NR-HARs from *D* involves generating all NR-HARs with a utility no less than a user-specified minimum utility threshold *min-util*, and a utility confidence no less than a user-specified minimum utility confidence threshold *min-uconf*.

Sahoo et al. [29] introduced a definition of non-redundant association rules in which $R_{1}:X_{1}\;\to Y_{1}$ and $R_{2}:X_{2}\;\to Y_{2}$ are two valid HARs in the utility-confidence framework. *R*_2_ is made redundant by *R*_1_ if $X_{2}\cup \;Y_{2}\;\subseteq X_{1}\;\cup \;Y_{1},\;R_{1}.\;utility\;\geq R_{2}.utility,\;$ $support\;\left(R_{1}\right)=Support\;\left(R_{2}\right)$, $X_{1}\;\subseteq X_{2},\;\mathrm{and}\;Y_{2}\;\subseteq Y_{1}$ where $R_{i}.utility$ is the utility of rule $R_{i},\;i=1,2$ and $support\;\left(R_{i}\right)=\;supp\left(X_{i}\cup \;Y_{i}\right)$. However, in most cases related to the mining of association rules, this definition should include a condition related to the confidence of the rules, as set out below.

**Definition** **1.** *Let*$R_{1}:X_{1}\;\to Y_{1}$*and*$R_{2}:X_{2}\;\to Y_{2}$*be two valid HARs in the utility-confidence framework. R_2_ is made redundant by R_1_ if*$X_{2}\cup \;Y_{2}\;\subseteq X_{1}\;\cup \;Y_{1},\;uconf(R_{1})\;\geq uconf(R_{2}),\;$$support\;\left(R_{1}\right)=Support\;\left(R_{2}\right)$*,*$X_{1}\;\subseteq X_{2},\;and\;Y_{2}\;\subseteq Y_{1}$.

## 4. Mining NR-HARs from a Lattice of High-Utility Itemsets

**Figure 1:**
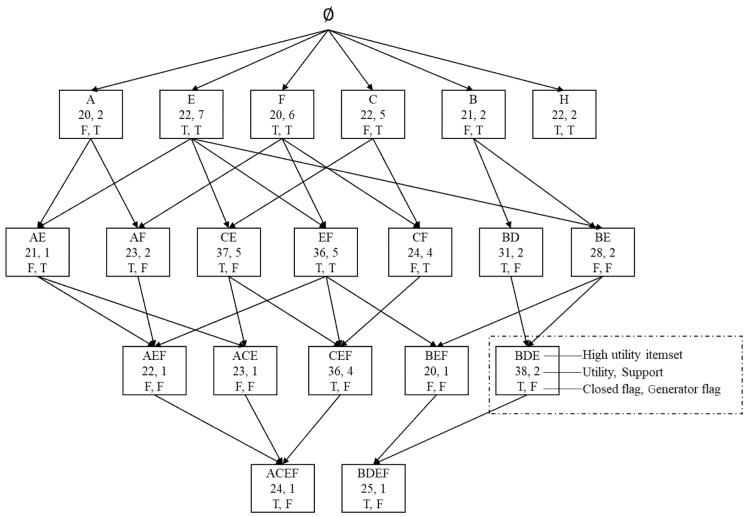
Lattice of HUIs with closed and generator itemsets.

The high-utility itemsets lattice (HUIL) structure is made up of several nodes, and there are parent–child relationships between each pair of nodes. Figure 1 shows the HUIL built from HUIs that are mined from above sample database in Table 1 and Table 2. The list of HUIs is presented in Table 3.

Each node represents an itemset, utility, support, “closed” flag and “generator” flag [36]. The utility and support values of the root node are equal to zero. If the value of the IsClosed flag for a node is true, the node represents a HUCI. Likewise, if the value of the IsGenerator flag for a node is true, the node represents a generator. The identity or name of a node is constructed by aggregating all the items in the itemset represented by the node.

### 4.1. Algorithm

The LNR-HAR algorithm is developed to traverse across the nodes in a HUCIL and mine all NR-HARs. Starting from the root, for each child node {*A*, *B*, *C, E*, *F*, *H*}, the FindNR-HARs method is triggered at line 3. The FindNR-HARs(latticeNode) method will search the rules with an antecedent *node* via the EnumerateNR-HARs method (line 7). If a *node* is a generator (*node*.IsGenerator = True) (line 6), then the FindNR-HARs method is called recursively (lines 10–12) for all child nodes of the *node*. In the EnumerateNR-HARs method, the queue data structure *Q* is initialized (line 14) with all child nodes of *childNode* (line 16), and each child node is tracked to avoid a collision using the *trackingList* collection variable (line 14). We then process each node *L_i_* taken from *Q*. If *L_i_* is a HUCI, rule $R:L_{c}.Itemset\;\to L_{i}.Itemset\;\setminus \;L_{c}.Itemset$ is used; if *R* has a utility confidence value (*R.uconf*) greater than or equal to *min-uconf* (line 24), *R* is added into the results (line 25–27). If *R* is valid, *Q* is enqueued in the list of child nodes of *L_i_* (lines 32–37). If *R* is invalid and *Q* still has itemsets, the algorithm continues to process the itemsets dequeued from *Q*. The details of algorithm 1 are described as follows.
**Algorithm 1**: LNR-HAR (HUCIL, *min-uconf*)**Input:** HUCIL, *min-uconf***Output:** Set of NR-HARs: *NRs***Methods**:**FindNR-HARsFromLattice()***1.* NRs= ∅*2.* **Foreach***childNode*∈*rootNode.Children**3.*           *FindNR-HARs(childNode)**4.* **End****FindNR-HARs**(*node*)*5.* **If***node.Flag* = *False* **then***6.*         **If** *node.IsGenerator* = *True* then*7.*                 *EnumerateNR-HARs(node)**8.*         **End***9.*         *node.Flag* = *True**10.*         **Foreach** *childNode* ∈ *node.Children**11.*                 *FindNR-HARs (childNode)**12.*         **End***13.* **End****EnumerateNR-HARs**(*node*)*14.* Q= ∅,  trackingList= ∅*15.* **Foreach***childNode*∈*node.Children**16.*         *Q.Enqueue(childNode)**17.*         *trackingList.Add(childNode)**18.* **End***19.* **While**Q  ∅*20.*         *L_i_* = *Q.Dequeue()**21.*         *ProcessChild* = *True**22.*         **If** *L_i_.isClosed* = *True* **then***23.*                 $R=\left\{node.Itemset\;\to L_{i}.Itemset\;\backslash \;node.Itemset\right\}$*24.*                 *u* = *CalculateConfidence (R)**25.*                 If uconf≥ *min-uconf* **then***26.*                         *R.conf* = *u**27.*                         **Add** *R into NRs**28.*                 **Else***29.*                         *ProcessChild* = *False**30.*                 **End***31.*           **End***32.*            **If** *ProcessChild* **then***33.*                 **Foreach** *L_c_* ∈ *L_i_.Chidren**34.*                         *Q.Enqueue(L_c_)**35.*                         *trackingList.Add(L_c_)**36.*                 **End***37.*           **End***38.* **End**

### 4.2. Illustrations

This section illustrates how the proposed LNR-HAR algorithm works to mine NR-HARs from the database given in Table 1 and Table 2. The LNR-HAR algorithm will generate a result of 10 NR-HARs as shown in Table 4.

Firstly, the result variable *NRs* is initialized to NULL. Then, the algorithm scans all children nodes of *rootNode*: *{A, B, C, E, F, H}*. For node *A*, which is a generator, the algorithm will call FindNR-HARs to check whether all of the rules formed by the antecedent *A* (*R*: A →X, X is child node of A) are valid. Below is a step-by-step description of the mining of rules from a lattice node *A* with *min-uconf* = 80%.

- Enter the FindNR-HARs method with itemset A (node A).
- A.Flag is False and A.IsGenerator is True, so the EnumerateNR-HARs (A) method is called.
- In EnumerateNR-HAR with A as an input parameter, declare Queue = ∅, MarkLNode = ∅.
- Enqueue *Q* and extend trackingList by the list of child nodes of *A*, Q={AE, AF}, trackingList={AE, AF}.
- Next, set $Q\;\neq \;\emptyset ,\;L_{i}=Q.Dequeue()=AE$.
- AE is not a HUCI. Push child nodes of AE into Q and trackingList. Q={ AF, ACEF}, trackingList={AE, AF, ACEF}.
- Next, set $Q\;\neq \;\emptyset ,\;L_{i}=Q.Dequeue()=AF.$
- Since AF.IsClosed = True (AF is an HUCI), $\;R_{A1}:A\;\to F,\;R.uconf=\;100\%>min-uconf\Rightarrow Add\;R_{A1}\;into\;RuleSet$. Since $R_{A1}$ is valid, the ProcessChild variable is True, and all child nodes of AF are inserted into the queue, Q={ACEF}, trackingList={AE, AF, ACEF}.
- Set $L_{i}=Q.Dequeue()=ACEF$.
- Since ACEF is a HUCI, $R_{A2}:A\;\to CEF,\;R.uconf=\;80\%\;\geq \;min-uconf\Rightarrow Add\;R_{A2}\;into\;RuleSet$. Then, child nodes of ACEF are pushed into Q and trackingList. Node ACEF has no child nodes, i.e., Q = ∅.
- FindNR-HARs is called recursively to process the child nodes {AE, AF} of A. The steps for finding rules from these child nodes are similar to those for the processing of node A.

The LNR-HAR algorithm will then carry out similar steps for the remaining child lattice nodes of the root node {E, F, C, B, H}. Since node H has no children (Figure 1), no rules are formed when processing node H.

Based on Definition 1, we introduce the HGB* algorithm by modifying the line 6 of the HGB algorithm [15] to achieve the same result, produce a more accurate set of results, as well as follow the best practice of association rule mining. The completed HGB algorithm with the modified sixth line is shown in algorithm 2.
**Algorithm 2:** HGB* (HUCI, *min-uconf*)**Input:** HUCI, min-uconf, min-util**Output:** Set of non-redundant high utility association rules *RuleSet***For** each itemset h ∈HUCI do**Set** Lma = ∅ //minimal antecedent**For** each $h^{\prime }\subseteq h$ in increasing order of size        **Set** Ltemp = ∅        **For** each g ∈HGh’ and g ≠h                **If** $\frac{luv\;\left(g,h\right)}{u\left(g\right)}\;\geq min-uconf$ **then** //*modified from HGB algorithm to obtain more accuracy set of non-redundant high utility association rules*                    Compute Lma=Lma ∪g                **else**                    Compute Ltemp=Ltemp ∪g                **End**          **End**          **For** each g ∈Ltemp                Compute A_1_ = {i_1_, i_2_, …., i_k_}, where each i_j_ $\in h^{\prime }\;\setminus g$                **For** (j = 1, A_j_ ≠ ∅;and (i ≤k);i++)                      **For** all l ∈ A_j_                            **If** ($\frac{luv\left(\left\{gl\right\},\;h\right)}{u\left(\left\{g\right\}\right)}\geq min-uconf$ and ∄ gs ∈Lma | gs ⊆{gl}) **then**                                  Remove all l’ ⊃{gl} from Lma                                  Compute Lma=Lma∪ {gl}                            **End**                    **End**                    Compute A_j+1_ = Apriorigen (A_j_, *min-util*)            **End**    **End**    **For** each gs ∈Lma            Compute R=gs →h  gs            Compute R.Utility=h.utility            Compute $R.uconf=\;\frac{luv\;\left(gs,\;h\right)}{u\left(gs\right)}$            Compute RuleSet=RuleSet ∪R    **End****End**

Although HGB* applies definition 1 to have more accurate data, its performance is still not better than LNR-HAR algorithm. The LNR-HAR algorithm extracts results from the lattice of HUIs having IsClosed and IsGenerator indicators, and then based on a flag, ProcessChild to determine finding rules with child nodes to be stopped or continued. This ProcessChild flag helps to save most of processing time. HGB or HGB* process all set of HUCIs, and for each HUCI these algorithms scan all subsets of each HUCI and generator to form a Lma list (a list of all the generators of a HUCI), and then process the Lma list to produce HARs. The whole process of HGB or HGB* needs a long time to finish as well as significant memory storage. The details of the comparison are described in the following section.

## 5. Experimental Results

HGB* algorithm is an extension of HGB algorithm to mine NR-HARs. However, HGB* aims to provide more accurate data since the problem of mining association rules should include a condition related to the confidence of the rules. The above change in algorithm 2, modifying the line 6 of the HGB algorithm [15], does not create the difference on performance in term of runtime and memory usage between HGB and HGB*. In this section, we compare the performance of the LNR-HAR algorithm with that of the HGB* in terms of mining NR-HARs. The experiments were implemented in the C# programming language using .Net framework 4.5. The testing machine was a sixth-generation quad-core 64 bit Core-i7 processor, clocked at 2.5 Ghz (6500U), running Windows 10 with 16 GB RAM. The testing datasets [40] are summarized in Table 5. The following datasets are common one that have often been used to evaluate the efficiency of algorithms. As detailed in below Table 5, Chess, Mushroom, and Accidents are dense datasets having small groups of items and a large number of items per transaction, while Retail and Chainstore are sparse datasets having large groups of items and fewer items per transaction. In terms of size, Chess and Mushroom are two small datasets, Accidents and Retail are medium-size datasets, and Chainstore is a large dataset.

We also executed our algorithm (LNR-HAR) on different datasets, with different values of *min-uconf* (between 60% and 90% in intervals of 10%), and selected *min-util* values according to each dataset. The results for the NR-HARs are reported in Table 6.

### 5.1. Runtime for Mining NR-HARs

We conducted testing and compared the performance between the LNR-HAR and HGB* algorithms on two kinds of dataset, sparse and dense. Sparse datasets have a large number of transactions compared to dense datasets, and the items appear in very few transactions. A large number of HUIs is generated from a dense dataset, in which there are also large numbers of HUCIs and generators. The LNR-HAR algorithm runs faster than the HGB* algorithm on dense datasets, although there is not much difference because both suffer from the problem of a large number of HUCIs and generators. The LNR-HAR algorithm also applies definition 1 to skip processing a large number of nodes. It operates more efficiently with the set of HUIs generated from a sparse dataset and produces results much faster than the HGB* algorithm. The following reports present the details of the runtime comparison on each dataset.

**Figure 2:**
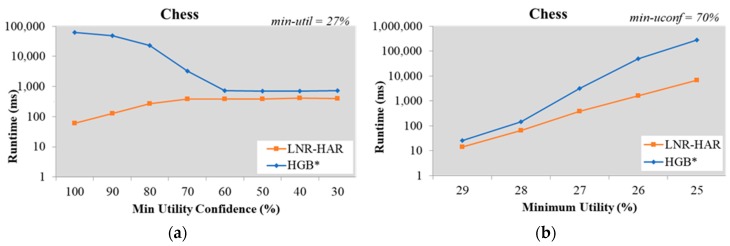
Runtime on the Chess dataset (**a**) with fixed *min-util* = 27% and various *min-ucon* and (**b**) with various *min-util* and fixed *min-uconf* = 70%.

For the same set of HUIs, the execution time of the LNR-HAR algorithm was reduced if the value of *min-uconf* was increased. In some testing datasets, such as Chess and Mushroom, there were few NR-HARs for a high value of *min-uconf* (greater than 60%). The runtime of HGB* for the mining of all NR-HARs, therefore, was increased if we increased *min-uconf* (Figure 2 and Figure 6).

When mining NR-HARs from the Chess dataset using the LNR-HAR algorithm with *min-util* = 27%, the execution time for *min-uconf* = 80% was 273.73 ms, for *min-uconf* = 90% it was 125.36 ms, and for *min-uconf* = 100% it was 61.02 ms. In contrast, with the HGB* algorithm it took 23.221 ms for *min-uconf* = 80%, 49.397 ms for *min-uconf* = 90%, and 61.932 ms for *min-uconf* = 100% (Figure 2a).

We also evaluated the performance on the Chess dataset using a constant value of *min-uconf* = 70% and several values for *min-util* ∈{29%, 28%, 27%, 26%, 25%}. The runtime for mining NR-HARs increased for both the HGB* and the LNR-HAR algorithms; however, LNR-HAR needed lesser time than HGB* (Figure 2b).

**Figure 3:**
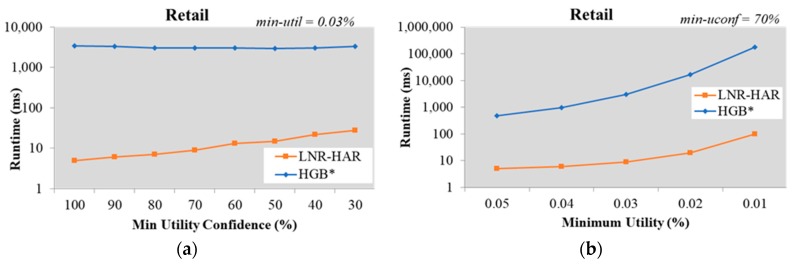
Runtime on the Retail dataset (**a**) with fixed *min-util* = 0.03% and various *min-uconf* and (**b**) with various *min-util* and fixed *min-uconf* = 70%.

Using a similar testing approach to that used for the Chess dataset, we kept *min-util* fixed at 0.03% and used different *min-uconf* values with the Retail dataset. The LNR-HAR algorithm needed much less time than the HGB* algorithm; while HGB* needed an average of 3,163 ms to extract all NR-HARs, LNR-HAR only took an average of 13 ms to complete this task (Figure 3a). We also increased the number of HUIs to evaluate the speed of LNR-HAR at a fixed value of *min-uconf* = 70%, as shown in Figure 3b. The runtimes for both the LNR-HAR and HGB* algorithms increased, although the former remained faster.

**Figure 4:**
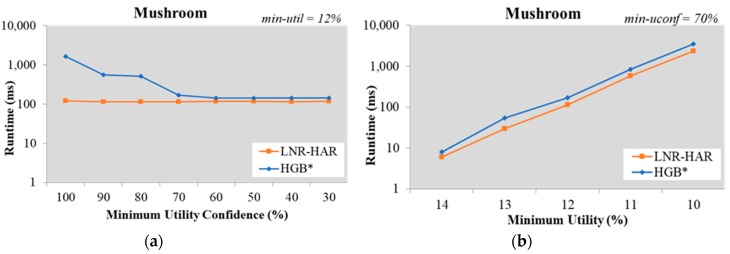
Runtime on the Mushroom dataset (**a**) with fixed *min-util* = 12% and various *min-uconf* and (**b**) with various *min-util* and fixed *min-uconf* = 70%.

For the Mushroom dataset, the execution time of LNR-HAR was 114.34 ms, and that of HGB* was 519.18 ms for *min-util* = 12% and *min-uconf* = 70% (Figure 4). This indicates that LNR-HAR performs better with regard to processing speed.

**Figure 5:**
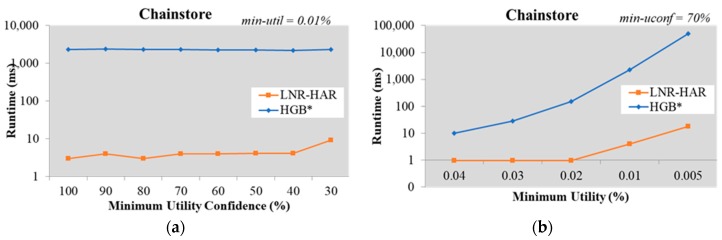
Runtime on the Chainstore dataset (**a**) with fixed *min-util* = 0.01% and various *min-uconf* and (**b**) with various *min-util* and fixed *min-uconf* = 70%.

**Figure 6:**
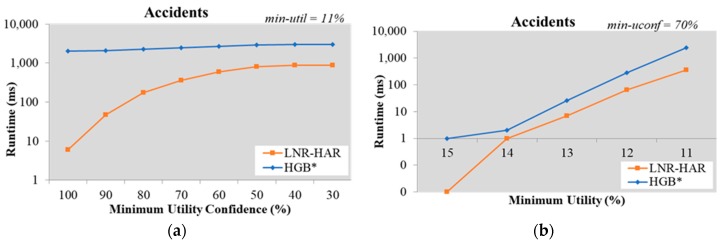
Runtime on the Accidents dataset (**a**) with fixed *min-util* = 11% and various *min-uconf* and (**b**) with various *min-util* and fixed *min-uconf* = 70%.

The results for the other datasets, namely Chainstore (Figure 5) and Accidents (Figure 6), also verified that LNR-HAR required a lower execution time than the HGB* approach.

The LNR-HAR algorithm showed its extremely good performance compared to the HGB* algorithm when both were applied to the Accidents dataset by keeping fixed *min-uconf = 70%* and inputting various *min-utility* ∈ {11%, 12%, 13%, 14%, 15%}. On the Accidents dataset, with *min-util = 15%*, the LNR-HAR algorithm only required 0.01 ms while HGB* required 0.97 ms to mine all NR-HARs having *min-uconf = 70%*. In other cases, where *min-util = 14%, min-uconf = 70%s*, the LNR-HAR algorithm needed 0.97 ms while HGB* took 2 ms; where *min-util = 11%*, *min-uconf = 70%*, the runtime for the LNR-HAR algorithm was only 15% of the runtime for HGB* (363 ms/2470 ms). Figure 6b shows the logarithmic scale chart indicating the performance comparison in term of runtime for mining NR-HARs from the Accidents dataset. Overall, the runtime to execute the LNR-HAR algorithm is much lesser than that required to execute the HGB* algorithm.

### 5.2. Memory Usage for Mining Non-Redundant Association Rules

**Figure 7:**
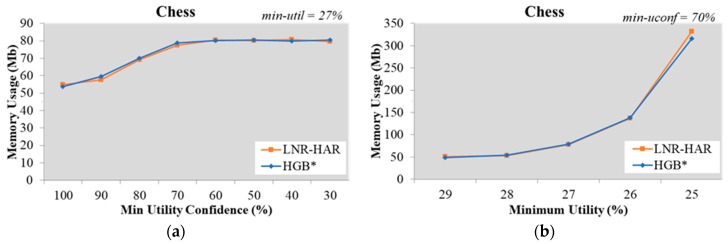
Memory usage on the Chess dataset (**a**) with fixed *min-util* = 27% and various *min-uconf* and (**b**) with various *min-util* and fixed *min-uconf* = 70%.

**Figure 8:**
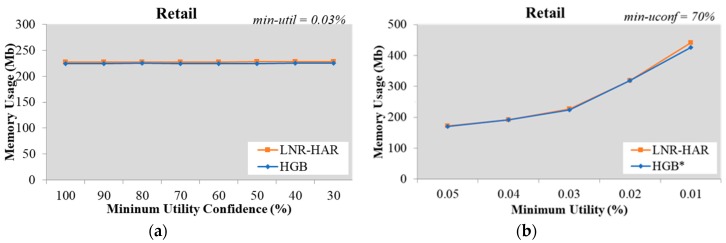
Memory usage on the Retail dataset (**a**) with fixed *min-util* = 0.03% and various *min-uconf* and (**b**) with various *min-util* and fixed *min-uconf* = 70%.

**Figure 9:**
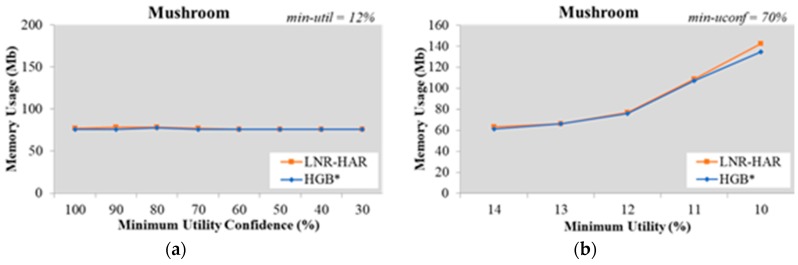
Memory usage on the Mushroom dataset (**a**) with fixed *min-util* = 12% and various *min-uconf* and (**b**) with various *min-util* and fixed *min-uconf* = 70%.

**Figure 10:**
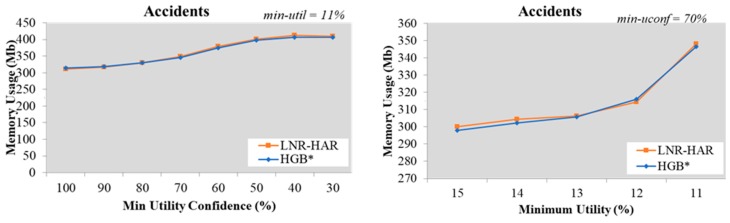
Memory usage on the Accidents dataset (**a**) with fixed *min-util* = 12% and various *min-uconf* and (**b**) with various *min-util* and fixed *min-uconf* = 70%.

**Figure 11:**
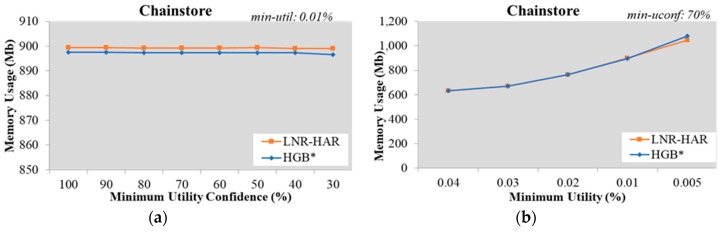
Memory usage on the Chainstore dataset (**a**) with fixed *min-util* = 12% and various *min-uconf* and (**b**) with various *min-util* and fixed *min-uconf* = 70%.

Since the mining of NR-HARs is based on HUCIs and generators, both LNR-HAR and HGB* have the same set of rules. The memory required to handle HUCIs, generators, and NR-HARs was also the same. Our experimental results, shown in Figure 7, Figure 8, Figure 9, Figure 10 and Figure 11, indicated that the memory usage required by both algorithms (LNR-HAR and HGB*) to mine all NR-HARs was approximately equal.

## 6. Conclusions

In this research, we present an efficient method for mining NR-HARs called LNR-HAR. The algorithm is based on the utility-confidence framework and the lattice concept, and can obtain the semantic relationships among HUIs. To the best of our knowledge, this is the first study of the mining of NR-HARs using a lattice structure. The outcome of this work is a new algorithm, LNR-HAR, and the modified algorithm HGB* from the HGB algorithm [15], in which the new LNR-HAR algorithm needs less execution time for the same memory usage as the HGB* algorithm. The approach can be integrated into various systems to quickly mine NR-HARs. The algorithm was tested on the popular datasets including both dense and sparse types, which could be generated from the systems based on IoT or sensor systems. We also compared the proposed algorithm to the previous research to evaluate the performance as well as the correctness. In future work, we intend to improve the memory usage of LNR-HAR algorithm and study ways of quickly generating HUIs, HUCIs, and generators, in order to rapidly extract HARs and NR-HARs. Besides, we intend to carry out research on providing non-redundant high-utility association rules as a representative training sets for machine learning algorithms to speed up the decision-making activities or predict the trends of customers.

## Figures and Tables

**Table 1:** Transaction database *D*.

Tid	Transaction
T1	B:4, D:1, E:6, F:2
T2	C:1, E:4, F:5
T3	A:4, C:1, E:5, F:1
T4	C:1, E:2, F:6
T5	B:3, C:1, E:1
T6	A:1, F:2, G:1
T7	C:1, E:1, F:4, G:1, H:1
T8	C:7, E:3
T9	H:10

**Table 2:** Items and weights (utilities).

Item	Utility
A	4
B	3
C	2
D	5
E	1
F	1
G	1
H	2

**Table 3:** List of high-utility itemsets (HUIs) from *D* with a *min-util* of 20.

Itemset	Utility	Itemset	Utility	Itemset	Utility
A	20	AF	23	ACE	23
B	21	BE	28	BEF	20
C	22	BD	31	BDE	38
E	22	CE	37	CEF	36
F	20	CF	24	ACEF	24
H	22	EF	36	BDEF	25
AE	21	AEF	22		

**Table 4:** Non-redundant high-utility association rules (NR-HARs) with *min-uconf* = 80%.

Rule	Confidence (%)	Utility	Support
A→F	100	23	2
F→E	90	36	6
A→CEF	80	24	2
B→DE	100	38	2
BEF→D	100	25	1
C→E	100	37	5
E→F	81	36	7
AE→CF	100	24	1
F→CE	80	36	6
CF→E	100	36	4

**Table 5:** Summary of the testing datasets.

Dataset	Transactions	Items	Size (MB)	Type
Chess	3196	75	0.63	Dense
Mushroom	8124	119	1.03	Dense
Accidents	340,183	468	63.1	Dense
Retail	88,162	16,470	6.42	Sparse
Chainstore	1,112,949	46,086	79.2	Sparse

**Table 6:** Number of NR-HARs from the testing datasets.

min-uconf (%)	Chess		Mushroom		Retail		Chainstore		Accidents	
	min-uti (%)	# of NR-HARs	min-uti (%)	# of NR-HARs	min-util (%)	# of NR-HARs	min-util (%)	# of NR-HARs	min-util (%)	# of NR-HARs
90	25	47,622	10	2405	0.01	1859	0.005	49	10	25,061
80	25	161,631	10	2774	0.01	5573	0.005	69	10	100,614
70	25	325,207	10	3178	0.01	12,819	0.005	143	10	232,272
60	25	439,584	10	3555	0.01	20,967	0.005	366	10	422,415
90	26	19,626	11	1376s	0.02	437	0.01	16	11	6411
80	26	61,469	11	1481	0.02	1441	0.01	19	11	23,911
70	26	111,304	11	1616	0.02	3751	0.01	31	11	51,700
60	26	139,006	11	1721	0.02	6629	0.01	67	11	83,388
90	27	8028	12	685	0.03	219	0.02	9	12	1657
80	27	22,945	12	707	0.03	664	0.02	11	12	5568
70	27	36,495	12	740	0.03	1733	0.02	12	12	11,332
60	27	39,614	12	757	0.03	3132	0.02	15	12	17,778
90	28	2788	13	334	0.04	149	0.03	5	13	367
80	28	7215	13	334	0.04	394	0.03	6	13	1024
70	28	9203	13	340	0.04	1025	0.03	6	13	1855
60	28	9,286	13	340	0.04	1862	0.03	7	13	2453
